# The PD-L1/PD-1 Axis Blocks Neutrophil Cytotoxicity in Cancer

**DOI:** 10.3390/cells10061510

**Published:** 2021-06-15

**Authors:** Olga Yajuk, Maya Baron, Sapir Toker, Tamir Zelter, Tanya Fainsod-Levi, Zvi Granot

**Affiliations:** Department of Developmental Biology and Cancer Research, Institute for Medical Research Israel Canada, Hebrew University Medical School, Jerusalem 91120, Israel; olga.yajuk@mail.huji.ac.il (O.Y.); mayagershko@gmail.com (M.B.); sapir.toker@mail.huji.ac.il (S.T.); tamir.zelter@mail.huji.ac.il (T.Z.); tanya.fainsod@mail.huji.ac.il (T.F.-L.)

**Keywords:** neutrophils, cancer, metastasis, PD-1, PD-L1

## Abstract

The PD-L1/PD-1 axis mediates immune tolerance and promotes tumor growth and progression via the inhibition of anti-tumor immunity. Blocking the interaction between PD-L1 and PD-1 was clinically shown to be beneficial in maintaining the anti-tumor functions of the adaptive immune system. Still, the consequences of blocking the PD-L1/PD-1 axis on innate immune responses remain largely unexplored. In this context, neutrophils were shown to consist of distinct subpopulations, which possess either pro- or anti-tumor properties. PD-L1-expressing neutrophils are considered pro-tumor as they are able to suppress cytotoxic T cells and are propagated with disease progression. That said, we found that PD-L1 expression is not limited to tumor promoting neutrophils, but is also evident in anti-tumor neutrophils. We show that neutrophil cytotoxicity is effectively and efficiently blocked by tumor cell-expressed PD-1. Furthermore, the blocking of either neutrophil PD-L1 or tumor cell PD-1 maintains neutrophil cytotoxicity. Importantly, we show that tumor cell PD-1 blocks neutrophil cytotoxicity and promotes tumor growth via a mechanism independent of adaptive immunity. Taken together, these findings highlight the therapeutic potential of enhancing anti-tumor innate immune responses via blocking of the PD-L1/PD-1 axis.

## 1. Introduction

Anti-tumor immune surveillance is an important feature of the immune system, where immune cells recognize and eliminate malignancies [1,2,3]. However, immune cells make up a significant proportion of the tumor microenvironment (TME) and were shown to contribute to tumor development and progression [4]. This dramatic change in immune function is mediated via various evasion mechanisms [5,6], one of which is the expression of immune checkpoint molecules by tumor cells. Under physiological conditions, the expression of immune checkpoint molecules regulates immune responses and prevents autoimmunity [7,8]. In cancer, the expression of these molecules enables blocking of anti-tumor immunity and promotes immune evasion [6].

The programmed cell death protein 1 (PD-1; also known as Pdcd1) receptor and its ligand, PD-1 ligand 1 (PD-L1), serve as an immune checkpoint pathway and are of significant clinical importance [9,10]. Tumor cells benefit from the expression of PD-L1 as its expression inhibits the proliferation and activation of cytotoxic T cells [11,12,13]. Interestingly, the expression of the inhibitory ligand, PD-L1, is not limited only to tumor cells in the TME [14]. Neutrophils in the TME and in the peritumoral tissue also express high levels of PD-L1 [15,16]. Tumor-infiltrating neutrophils were shown to play important, yet multifaceted and sometimes opposing roles [17]. The tumor-promoting properties of neutrophils were widely described as they have the ability to initiate tumorigenesis [18], enhance tumor progression via direct stimulation of tumor cell proliferation [19], enhance angiogenesis [20,21], suppress anti-tumor immune effector cells [22,23], and promote metastatic spread [24,25,26]. In contrast, studies have shown that neutrophils may also possess anti-tumor features, as they can kill tumor cells via the secretion of H_2_O_2_ [27,28]. The role of PD-L1^pos^ neutrophils is associated with a tumor-promoting phenotype as they were found to suppress cytotoxic T cells, thereby enhancing disease progression and shortening patient survival [15,23].

Although the expression of PD-L1 is mainly attributed to tumor cells and PD-1 expression is attributed to T cells, the expression pattern of PD-L1 and PD-1 in cancer is more complex [14,29]. A study by Kleffel et al. [30] showed that murine and human melanomas contain PD-1-expressing cancer subpopulations and demonstrate that melanoma PD-1 cell-intrinsic signaling promotes tumorigenesis. Although the role of PD-L1 on neutrophils is not fully understood, this observation raises the possibility that PD-L1^pos^ neutrophils can directly interact with PD-1^pos^ tumor cells. In this study, we examined the role played by the PD-L1/PD-1 axis in regulating neutrophil anti-tumor cytotoxicity. We found that neutrophil PD-L1 not only acts to suppress T cell responses, but may also mediate the inhibition of neutrophil cytotoxicity. Blocking the PD-L1/PD-1 interaction enhances tumor cells susceptibility to neutrophil cytotoxicity. In a previous study [28], we showed that neutrophils possess an anti-metastatic role when encountering tumor cells in the premetastatic niche. In line with these observations, we found that tumor cell expression of PD-1 has a significant impact on neutrophil cytotoxicity, primary tumor growth, and metastatic progression. Our observations provide a wider scope to the role PD-L1 plays on neutrophils in cancer, and highlight the therapeutic potential of blocking the PD-L1/PD-1 axis not only in propagating anti-tumor T cell responses, but also in facilitating neutrophil cytotoxicity.

## 2. Materials and Methods

### 2.1. Animals

Five to six week old BALB/c, C57Bl6, NOD-SCID, and NSG mice were purchased from the Harlan (Jerusalem, Israel). All experiments involving animals were approved by the Hebrew University’s Institutional Animal Care and Use Committee (IACUC).

### 2.2. Cell Lines

Mouse breast cancer cells, 4T1 and AT3, were purchased from the ATCC and cultured in DMEM containing 10% heat-inactivated fetal calf serum (FCS). The 4T1 and AT3 cells were transduced with a lentiviral vector (pLVX-Luc, MigR1-Luc, or the triple-modality reporter gene TGL [31]) to stably express firefly luciferase. PD1kd 4T1 and AT3 cells were generated by lentiviral transduction with PD1-specific shRNAs (TRCN0000097670-74) from Sigma (St. Louis, MO, USA). Control cells were transduced via an empty vector (pLKO). Killing assay neutrophils and tumor cells were cultured in an RPMI containing 2% heat-inactivated fetal calf serum (FCS). For tumor engraftment, 1 × 10^6^ cells were orthotopically injected into the mammary fat pad. Tumor volume was determined by caliper measurement of tumor length and width (tumor vol. = length × width^2^ × 0.52).

### 2.3. Plasmids 

*Retroviral MigR1-Luc vector*: the open reading frame of the firefly luciferase was prepared from pLVX-luciferase using the forward primer 5′-CAG TCC GCT CGA GGC CGC CAC CAT GGA AGA CGC CAA AAA CAT AAA G-3′ containing a XhoI restriction site, Kozac sequence followed by the ATG initiation code and reverse primer 5′-ACT TCC GGA ATT CTT ACA CGG CGA TCT TTC CGC CC-3′ containing an EcoRI restriction site, and the stop codon and Phusion Flash High-Fidelity PCR master mix (Thermo Scientific). The amplified luciferase PCR product was digested by XhoI and EcoRI and inserted into the XhoI/EcoRI site of the MigR1 plasmid.

*Mouse PD1 expression vector*: mouse PD1 was prepared by PCR on cDNA from 4T1 breast cancer cells using a Phusion Flash High-Fidelity PCR master mix (Thermo Scientific, Waltham, MA, USA) and the following primer pair F 5′-ACT TCC GGA ATT CGC CGC CAC CAT GTG GGT CCG GCA GGT ACC CTG GTC-3′ containing an EcoRI site and nt 67–89 of Pdcd1 (NM_008798.2), and R 5′-AAG GAA AAA AGC GGC CGC TCA AAG AGG CCA AGA ACA ATG TCC-3′ containing a NotI site and nt 907–930 of Pdcd1. The EcoRI/NotI-cut PCR product was mixed with annealed Flagx3 primers having sticky ends of EcoRI and NotI: F 5′-ACT TCC GGA ATT CGC CGC CAC CAT GTG GGT CCG GCA GGT ACC CTG GTC-′3 and R 5′-AAG GAA AAA AGC GGC CGC TCA AAG AGG CCA AGA ACA ATG TCC-′3; and inserted into the EcoRI/NotI site of the pLVX-Puro vector. 

*Mouse PD1 CRSPR-Cas9 vector*: to generate PD-1 knockout cells, we used the Cas9 lentiGuide-Puro vector (Addgene 52963) with the following primers, F 5′-CAC CGG CAC CCC AAG GCA AAA ATCG-3′ and R 5′-AAA CCG ATT TTT GCC TTG GGG TGCC-3′, inserted into the plasmid digested with BsmBI. All plasmids were sequenced at the Center for Genomic Technologies at the Givat Ram Campus, the Hebrew University of Jerusalem.

### 2.4. Neutrophil Purification

The neutrophils were purified from orthotopically injected (mammary fat pad) 3-week-old, 4T1 tumor-bearing mice as described previously [32]. In brief, whole blood was collected by cardiac puncture using a heparinized (Sigma) syringe. The blood was diluted with 5 volumes of PBS containing 0.5% BSA and subjected to a discontinuous Histopaque (Sigma) gradient (1.077 and 1.119). NDN were collected from the 1.077–1.119 interface. LDN were collected from the plasma-1.077 interface. Red blood cells (RBCs) were eliminated by hypotonic lysis. Neutrophil purity and viability were determined visually and were consistently >98% for NDN. PD-L1 positive and negative neutrophils were isolated using EasySep^TM^ PE positive selection kit (STEMCELL) according to the manufacturer′s instructions.

### 2.5. Primary Tumor and Metastasis Formation In Vivo Assay

Immunodeficient mice (NSG or NOD-SCID) were injected, in the mammary fat pad, with 1 × 10^6^ control, PD1 over-expressing or PD-1ko tumor cells. The tumors were measured weekly. On day 27 post-tumor engraftment, the mice were euthanized and the lungs were excised for an analysis of the metastatic spread. The lungs were sectioned into 100 μm intervals and stained with H&E. The number of metastatic foci was determined by histological examination. The data represents the average tumor size of at least 5 mice per group.

### 2.6. qPCR

Total RNA was isolated with TRI-Reagent (Sigma) according to the manufacturer’s instructions. The RNA was reverse transcribed into cDNA using the AB high capacity cDNA kit (Applied Biosystems, Waltham, MA, USA). Two nanograms of converted cDNA was used for each reaction, using the Kapa Sybr-Green Master Mix (Kapa Biosystems, Wilmington, MA, USA) and 300 nM of forward/reverse primer set. Analyses were done using the CFX384 BioRad real-time PCR. GAPDH was used as a reference gene. The following primer sequences were used for gene expression analyses: PD1-F 5′-AGC GTA TCT GCT GTC CTT CTG-3′, PD1-R 5′-GGT TCC CTT TAT TGC CCT AGT T-3′, GAPDH-F 5′-GCC TTC CGT GTT CCT ACC-3′, and GAPDH-R 5′-CCT GCT TCA CCA CCT TCT T-3′.

### 2.7. Peritonitis

BALB/c mice were injected intraperitoneally (i.p.) with 1 mg of zymosan A from *Saccharomyces cerevisiae* (Sigma), diluted in 1 mL of sterile PBS. Cells were retrieved using peritoneal lavage at four different time points (3, 24, 48, and 72 h post i.p. injection). After purification and red blood cell (RBC) hypotonic lysis, the cell pellet was stained for PD-L1 and Ly6G and measured by flow cytometry.

### 2.8. ROS Production Assay

Purified PD-L1^pos^ and PD-L1^neg^ neutrophils were plated (2 × 10^5^) in 180 µL of HBSS (Biological Industries) in white 96 flat-bottom wells (Corning) containing 50 µM luminol (Sigma). ROS production (chemiluminescence) was measured using a Tecan F200 microplate luminescence reader. 

### 2.9. In Vitro Killing Assay

Luciferase-labeled tumor cells (1 × 10^4^/well) were plated in 100 µL RPMI-1640 with 2% FCS in white 96 flat-bottom wells (Corning). After a 24 h incubation period, purified neutrophils (1 × 10^5^/well in 100 µL) were added to the plated tumor cells and co-cultured overnight in the presence or absence of anti-PD-L1 2 µg (BioLegend). Following overnight incubation, luciferase activity was measured using a Tecan F200 microplate luminescence reader. The extent of killing was determined by the ratio between tumor cells cultured alone and tumor cells co-cultured with neutrophils. The in vitro experiments were repeated at least three times. 

### 2.10. BrdU Labeling

Tumor bearing mice were injected ip with 100 µL of BrdU solution (10 mg/mL in sterile PBS, BD Pharmingen, San Diego, CA, USA). The incorporation of BrdU was detected using the FITC BrdU Flow Kit (BD Pharmingen) and analyzed by flow cytometry.

### 2.11. Antibodies

Antibodies included mouse α-PD-L1 (BioLegend), α-mouse PD-L1-PE (BioLegend), α-mouse PD1-APC (BioLegend), α-mouse Ly6G-VioBlue (TONBO Biosciences), and α-mouse Ly6G (clone 1A8, BioXCell).

### 2.12. Statistical Analysis

For the studies comparing differences between two groups, we used unpaired Student’s t-tests. The differences were considered significant when *p* < 0.05. Data are presented as mean ± SEM.

## 3. Results

### 3.1. Immature Low-Density Neutrophils Express Higher Levels of PD-L1

**Figure 1:**
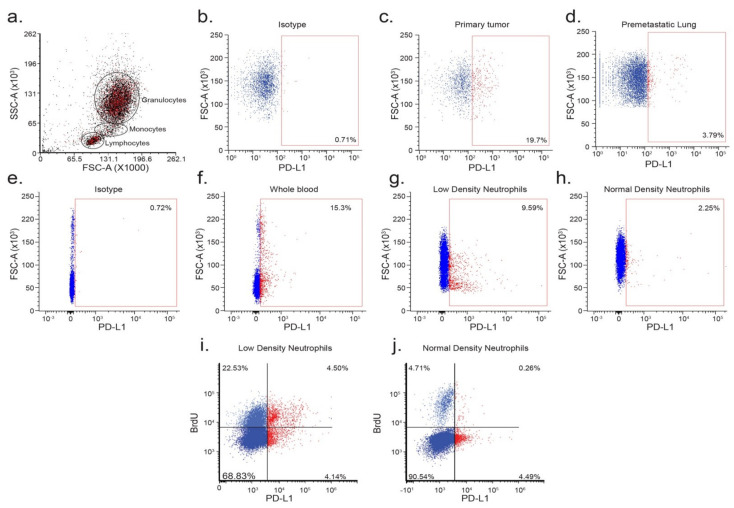
PD-L1^pos^ neutrophils are associated with an immature, low-density phenotype. The (**a**) FACS analysis of PD-L1^pos^ cells (red) in whole blood from a tumor-bearing mouse. FACS analysis of isotype control (**b**) and PD-L1 expression in Ly6G^+^ neutrophils isolated from the primary tumor (**c**), the premetastatic lung (**d**), and the circulation (**e**) of a tumor-bearing mouse. Red gate represents the PD-L1^pos^ subpopulation. The FACS analysis of PD-L1 expression in Ly6G^+^ neutrophils in whole blood (**f**), isolated low-density neutrophils (**g**), and isolated normal-density neutrophils (**h**) from a tumor-bearing mouse. Gating was determined on isotype control staining of Ly6G^+^ cells (**e**). Representative FACS analyses of BrdU staining of circulating Ly6G^+^ low-density neutrophils (**i**) and normal-density neutrophils (**j**) from a 4T1 tumor-bearing mouse.

The PD-L1/PD-1 immune checkpoint is well characterized in the context of cancer, where PD-L1-expressing tumor cells were shown to functionally inhibit the cytotoxicity of PD-1-expressing T cells. Furthermore, the blocking of the PD-L1/PD-1 interaction resulted in the maintenance of T cell anti-tumor cytotoxicity and the consequent reduction in tumor mass. Although the expression of PD-L1 is mainly attributed to tumor cells, PD-L1 was also expressed by various immune cells including lymphocytes, monocytes, and granulocytes (Figure 1a).

We previously showed that neutrophil anti-tumor activity is context dependent and can be either manifested or inhibited in different tumor microenvironments (circulation, primary tumor, premetastatic and metastatic sites). Specifically, we showed that tumor associated neutrophils (TAN) do not exhibit anti-tumor functions, whereas circulating neutrophils or neutrophils associated with the premetastatic niche exhibit high anti-tumor cytotoxicity [33]. To gain insight into the role PD-L1 plays in regulating neutrophil function, we evaluated the expression of PD-L1 on neutrophils isolated from the circulation, the primary tumor, and the premetastatic lung. We showed that PD-L1 ^pos^ neutrophils made up a significant proportion (~20%) of primary TAN (Figure 1b,c). PD-L1^pos^ neutrophils may also be detected in the premetastatic lung, albeit to a lesser extent (~4%, Figure 1d). We further showed that in the circulation, ~15% of Ly6G^+^ neutrophils were PD-L1^pos^ (Figure 1e,f). As we showed before, neutrophils may be divided into normal and low-density subsets (NDN and LDN, respectively, [34]). Since LDN were shown to possess pro-tumorigenic properties, we hypothesized that PD-L1 expression on neutrophils may be associated with this specific neutrophil subset. Indeed, while NDN consist of a small PD-L1^pos^ subset (~2%), a significantly higher proportion of LDN were PD-L1^pos^ (~10%; compare Figure 1g,h). LDN are heterogeneous and consist of both mature and immature neutrophils. Since immature neutrophils were shown to possess pro-tumor properties [34,35], we sought to determine whether PD-L1^pos^ neutrophils exhibit a more immature phenotype. To this end, we treated tumor-bearing mice with a single dose of BrdU and assessed the fraction of BrdU^+^ neutrophils 24 h following BrdU administration (a time point where only immature cells are BrdU^+^, [34]). In the low-density fraction, 16.6% of the BrdU^+^ (immature) neutrophils were PD-L1^pos^, compared with only 5.6% of mature (BrdU^−^) neutrophils (Figure 1i). The NDN fraction, which contained a small subpopulation of immature cells, consisted of a low percentage of PD-L1^pos^ neutrophils (~5%), with no significant difference between the mature and immature subpopulations (Figure 1j). 

**Figure 2:**
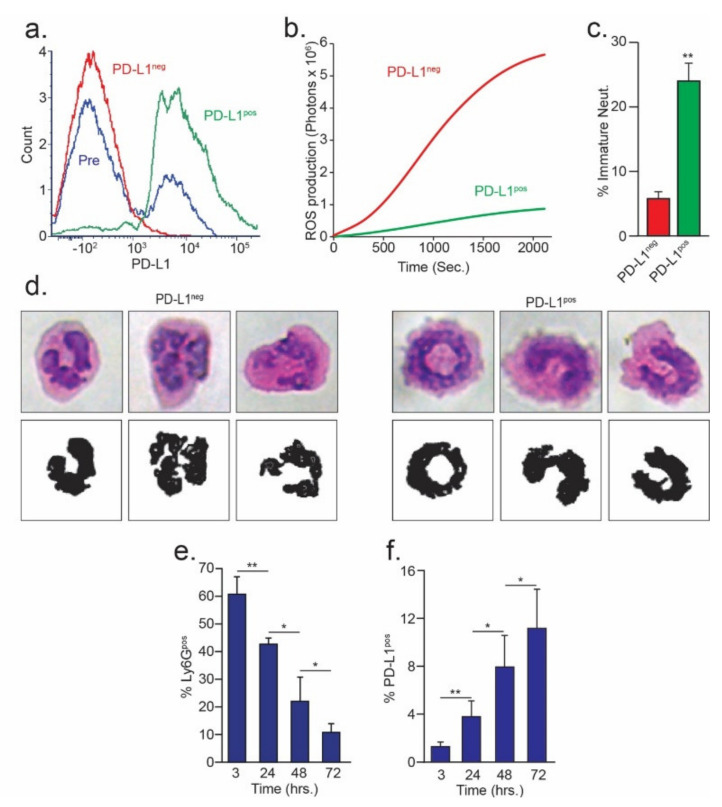
PD-L1^pos^ neutrophils are associated with the resolution phase in inflammation. (**a**) FACS analysis of PD-L1 expression in presorted neutrophils (Pre), PD-L1^neg^ neutrophils (red), and PD-L1^pos^ neutrophils (green); (**b**) spontaneous ROS production in PD-L1^neg^ (red) and PD-L1^pos^ (green) neutrophils; (**c**) percent of immature cells in PD-L1^neg^ (red) and PD-L1^pos^ (green) neutrophil subsets; (**d**) representative images of H&E staining of PD-L1^pos^ and PD-L1^neg^ neutrophils (top) and corresponding nuclear silhouettes (bottom); (**e**) time-dependent changes in the fraction of peritoneal neutrophils (of all WBC) in self-resolving peritonitis; and (**f**) time dependent changes in the fraction of peritoneal of PD-L1^pos^ neutrophils (of all Ly6G^+^ cells) in self-resolving peritonitis. * *p* < 0.05, ** *p* < 0.01.

To gain insight into their functional differences, we isolated circulating PD-L1^pos^ and PD-L1^neg^ neutrophils from tumor-bearing mice (Figure 2a). We found that oxidative burst (the production of H_2_O_2_) was significantly higher in the PD-L1^neg^ neutrophils (Figure 2b). In addition, we noted that PD-L1^pos^ cells contained a larger proportion of morphologically immature neutrophils (i.e., banded vs. fragmented nuclei, Figure 2c,d). This led us to hypothesize that, in general, PD-L1^neg^ cells may be viewed as pro-inflammatory, whereas PD-L1^pos^ neutrophils may be viewed as anti-inflammatory. To test whether PD-L1^neg^ neutrophils exhibit a more pro-inflammatory phenotype compared with PD-L1^pos^ neutrophils, we used the zymosan A-induced mouse model of peritonitis. This model simulates self-resolving acute inflammation, where peritoneal neutrophil numbers peak at 3 h post-zymosan A injection and gradually decrease over a period of 72 h. We previously showed that the phenotype of the peritoneal neutrophils changed from pro- to anti-inflammatory towards resolution of the inflammatory response [34]. Accordingly, we used this model to assess whether the anti-inflammatory, presumably more suppressive neutrophils expressed higher levels of PD-L1. As expected, the percentage of peritoneal Ly6G^+^ neutrophils gradually decreased towards resolution (Figure 2e). Notably, although the percentage of neutrophils gradually decreased, the fraction of PD-L1^pos^ neutrophils increased with time (Figure 2f). Taking into account that neutrophils acquire an anti-inflammatory phenotype towards resolution of inflammation [34], the gradual increase in the proportion of PD-L1^pos^ neutrophils towards resolution may indicate a possible immunosuppressive role for the PD-L1^pos^ neutrophil subpopulation. 

### 3.2. PD-1-Expressing Tumor Cells Inhibit Neutrophil Cytotoxicity via the PD-L1/PD-1 Axis

**Figure 3:**
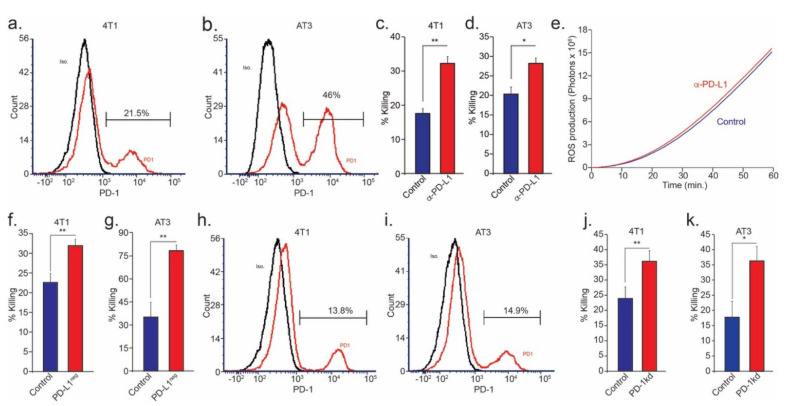
Blocking the PD-L1/PD-1 axis increases tumor cell susceptibility to neutrophil cytotoxicity. The FACS analysis of PD-1 expression in 4T1 (**a**) and AT3 (**b**) tumor cells; neutrophil mediated killing of 4T1 (**c**) and AT3 (**d**) tumor cells in the absence (cont.) or presence of PD-L1 blocking antibody; (**e**) spontaneous neutrophil ROS production in the presence (red) or absence (blue) of PD-L1 blocking antibody; neutrophil mediated killing of 4T1 (**f**) and AT3 (**g**) tumor cells with unsorted neutrophils (cont.) or PD-L1^neg^ neutrophils; FACS analysis of PD1 expression in PD-1kd 4T1 (**h**) and AT3 (**i**) cells tumor (red); and neutrophil-mediated killing of control and PD-1kd 4T1 (**j**) and AT3 (**k**) tumor cells. * *p* < 0.05, ** *p* < 0.01.

The notion that PD-L1 expression on neutrophils promotes pro-tumor features was explored and established in previous studies [36]. However, the effect of the PD-L1/PD-1 axis on neutrophil anti-tumor cytotoxicity was never examined. To determine the direct consequence of the PD-L1/PD-1 axis on neutrophils cytotoxicity, we assessed the expression levels of PD-1 on both neutrophils and tumor cells. We found that, while neutrophils do not express PD-1 (Figure S1), a significant subpopulation of both 4T1 and AT3 breast cancer cell lines do express PD-1 (Figure 3a,b). Importantly, we found that in a co-culture setting, specific blocking of PD-L1 enhanced the susceptibility of both 4T1 and AT3 cells to neutrophil cytotoxicity (Figure 3c,d). Notably, treating a neutrophil with a PD-L1 antibody did not affect ROS production (Figure 3e). This observation suggests that PD-1 expression on tumor cells can inhibit neutrophil cytotoxicity via the PD-L1/PD-1 axis. However, this experimental platform suffers from two significant flaws: first, the PD-L1 antibody can directly affect PD-L1 expressing tumor cells, and second, the addition of an antibody to the culture may result in neutrophil killing of tumor cells via antibody-dependent cellular cytotoxicity (ADCC). To overcome these difficulties, we isolated a pure population of PD-L1^neg^ neutrophils and tested their cytotoxic capacity. Using this approach, we were able to simultaneously eliminate possible PD-L1/PD-1 interactions and avoid the presence of an antibody in the culture. We found that when co-cultured with tumor cells, PD-L1^neg^ neutrophils exhibited a significantly higher cytotoxic ability compared with the mixed population (Figure 3f,g), independently corroborating the results obtained by the PD-L1 blocking antibody. Finally, we used PD-1 specific shRNA to knockdown PD-1 expression in both 4T1 and AT3 cells (Figure 3h,i). We then co-cultured control and PD-1kd cells with cytotoxic neutrophils and found that PD-1kd cells were more susceptible to neutrophil cytotoxicity (Figure 3j,k). Taken together, our observations suggest that tumor cell PD-1 limits neutrophil cytotoxicity and that PD-L1^neg^ neutrophils have enhanced cytotoxic potential.

### 3.3. Tumor Cell PD-1 Inhibits Neutrophil Cytotoxicity and Increases Metastatic Potential

**Figure 4:**
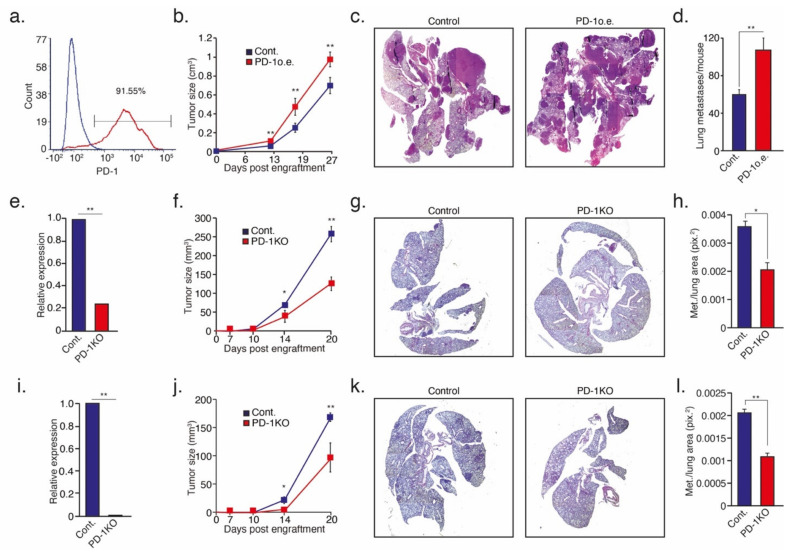
The effect of modulating tumor PD-1 expression on tumor growth and metastatic spread. The (**a**) FACS analysis of PD-1 overexpression in 4T1 tumor cells; (**b**) tumor growth of orthotopically injected control and PD-1 overexpressing 4T1 cells; (**c**) representative H&E staining of lungs from mice injected with control or PD-1 overexpressing 4T1 cells; (**d**) total number of spontaneous metastases in control and PD-1 overexpressing 4T1 tumor-bearing mice; (**e**) relative expression of PD-1 in control and PD-1ko 4T1 cells; (**f**) tumor growth of orthotopically injected control and PD-1ko 4T1 cells; (**g**) representative H&E staining of lungs from mice injected with control or PD-1ko 4T1 cells; (**h**) number of metastases per lung area in control and PD-1ko 4T1 tumor-bearing mice; (**i**) relative expression of PD-1 in control and PD-1ko AT3 cells; (**j**) tumor growth of orthotopically injected control and PD-1ko AT3 cells; (**k**) representative H&E staining of lungs from mice injected with control or PD-1ko AT3 cells; and (**l**) number of metastases per lung area in control and PD-1ko AT3 tumor-bearing mice. In all experiments *n* ≥ 5, and data is presented as average ± SE. * *p* < 0.05, ** *p* < 0.01.

To gain insight into the consequences of PD-1 expression on neutrophil function in the context of cancer, we tested how tumor growth and metastatic progression are affected by the modulation of the tumor cell expression of PD-1. To this end, we used two complementary approaches of both overexpressing and knocking out PD-1 in tumor cells. We started with testing the consequences of overexpressing PD-1 in 4T1 cells (Figure 4a). Notably, PD-1 plays a critical role in mediating the interaction between tumor cells and lymphocytes, which may interfere with proper interpretation of the experiments. To overcome this difficulty, we assessed tumor growth and metastatic progression in the orthotopically (mammary fat pad) injected control vs. the PD-1 overexpressing 4T1 tumor cells in NOD-SCID mice (practically devoid of adaptive immunity). Our data showed that PD-1 overexpressing 4T1 tumors exhibited a more aggressive phenotype as they grew faster (Figure 4b) and had a significantly higher metastatic load (Figure 4c,d) compared to control tumors. Since these mice lacked adaptive immunity, these observations highlight the inhibitory effect of the PD-L1/PD-1 axis on innate immune cells. While this observation supports our hypothesis, the natural background expression of PD-1 may still play a role in these settings. To get a clearer view of the role tumor cell PD-1 plays in regulating neutrophil function we used CRISPR-Cas9 to generate a PD-1ko 4T1 cell line (Figure 4e). Of note, repeated efforts to generate complete knockout of PD-1 in 4T1 always resulted in residual PD-1 transcript (Figure 4e). Still, when introduced into the mammary fat pad of immune-deficient NSG mice, PD-1ko resulted in a dramatic reduction in primary tumor growth (Figure 4f) and metastatic spread to the lungs (Figure 4g,h). To further support this observation, and to provide a broader scope to our results, we repeated this experiment in the AT3 cell where the knocking out of PD-1 was complete (Figure 4i). Much like 4T1 cells, PD-1ko AT3 tumors in NSG mice grew significantly slower (Figure 4j) and generated fewer metastases (Figure 4k,l). 

**Figure 5:**
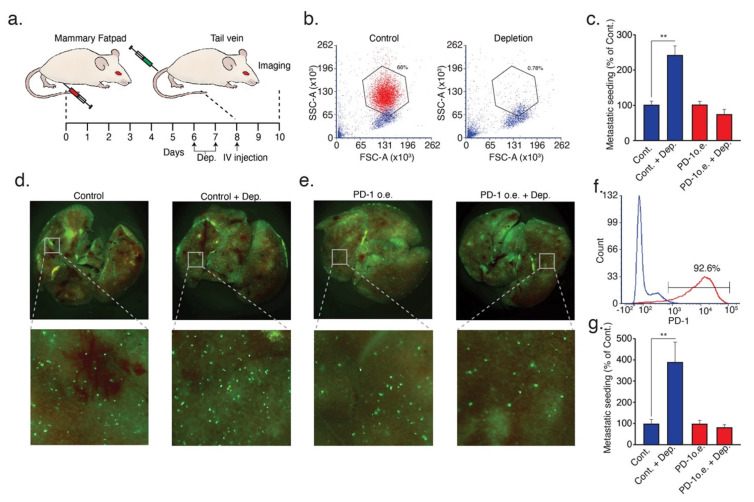
PD-1 overexpression protects tumor cells from neutrophil-mediated elimination at the premetastatic lung. (**a**) The experimental metastatic seeding assay-female NOD-SCID mice were orthotopically (mammary fat pad) injected with control (4T1 or AT3) tumor cells. Starting on day six, the mice were injected daily (arrows) with either control IgG or neutrophil depleting anti-Ly6G antibody i.p. On day eight, the mice were injected with 5 × 10^5^ GFP^+^ control or PD-1 overexpressing cells (4T1 or AT3) via the tail vein. On day 10, the number of lung-associated GFP^+^ cells were analyzed by fluorescent microscopy; (**b**) FACS analysis of circulating Ly6G^+^ neutrophils in control and neutrophil-depleted tumor-bearing mice; (**c**) average number of lung-associated GFP^+^ control and PD-1 overexpressing 4T1 tumor cells in control and neutrophil-depleted mice (*n* = 5); (**d**) representative images of lung-associated control GFP^+^ cells in control mice (control) and neutrophil-depleted mice (control + dep.); (**e**) representative images of lung-associated PD-1 overexpressing GFP^+^ cells in control mice (PD-1 o.e.) and neutrophil-depleted mice (PD-1 o.e. + dep.); (**f**) FACS analysis of PD-1 overexpression in AT3 tumor cells; and (**g**) average number of lung-associated GFP^+^ control and PD-1 overexpressing AT3 tumor cells in control and neutrophil-depleted mice (*n* = 5). ** *p* < 0.01.

Since these mice lacked adaptive immunity, these observations highlight the inhibitory effect of the PD-L1/PD-1 axis on innate immune cells. However, since this effect on tumor growth and metastatic progression may be mediated by cells other than neutrophils, we tested the consequences of neutrophil depletion on metastatic seeding of control and PD-1 overexpressing tumor cells. First, we orthotopically injected NOD-SCID mice with control 4T1 tumor cells in order to generate a primary tumor and prime the immune system. Next, we injected the control or PD-1 overexpressing 4T1^GFP+^ tumor cells via the tail vein to control for neutrophil-depleted tumor-bearing mice (Figure 5a). Corroborating previous observations, neutrophil depletion (Figure 5b) enhanced the seeding of control 4T1^GFP+^ tumor cells in the lungs (Figure 5c,d). In contrast, neutrophil depletion had no significant effect on the seeding of PD-1 overexpressing 4T1^GFP+^ tumor cells (Figure 5c,e). To broaden the scope of these observations, we implemented the same approach and overexpressed PD-1 in AT3 cells (Figure 5f). As in 4T1 cells, neutrophil depletion increased the seeding of control tumor cells in the lungs (Figure 5g), but had no effect on metastatic seeding of PD-1 overexpressing cells (Figure 5g). These observations suggest that PD-1 expression protects tumor cells from neutrophil cytotoxicity and promotes metastatic seeding.

Collectively, our data identified a new role for tumor cell expressed PD-1 in regulating neutrophil function via neutrophil-expressed PD-L1. We showed that PD-L1+ neutrophils were less cytotoxic than PD-L1 neutrophils and that blocking PD-L1/PD-1 interaction enhanced neutrophil anti-tumor cytotoxicity. This contribution is manifested when PD-1 overexpressing or PD-1ko tumor cells were introduced into mice which lacked adaptive immunity, but had abundant neutrophils. In these mice, where T cells no longer played a role, we saw that the loss of PD-1 limited tumor growth and metastasis, whereas over expressing PD-1 led to enhanced tumor growth and metastatic progression.

## 4. Discussion

**Figure 6:**
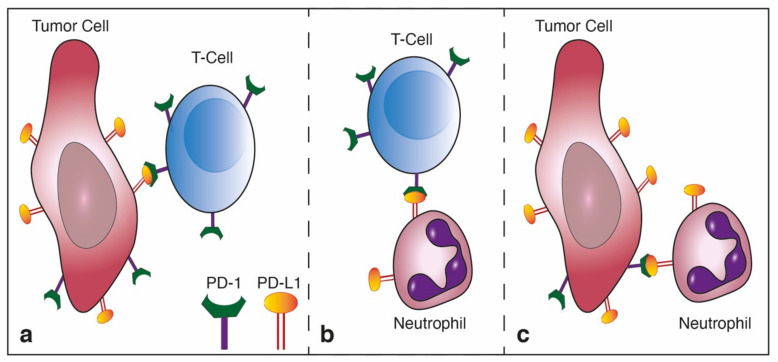
Mechanisms of tumor immune evasion mediated by the PD-L1/PD-1 axis. (**a**) Tumor cell expressed PD-L1 serves as a ligand for PD-1 expressed on cytotoxic T cells. This interaction leads to blocking of anti-tumor T cell responses. Targeting this interaction serves as the basis for anti-cancer immunotherapy; (**b**) Neutrophils possessing immunosuppressive properties express PD-L1, which serves as a ligand for PD-1 expressed on cytotoxic T cells, blocking T cell anti-tumor responses, and (**c**) tumor cell expressed PD-1 interacts with PD-L1 expressed by neutrophils, blocking cytotoxic anti-tumor neutrophil responses.

In recent years, our understanding of the multifaceted roles neutrophils play in the tumor microenvironment has expanded significantly, and we know they may either act to promote or limit tumor growth and progression. The present study identifies an important role of the PD-L1/PD-1 axis in regulating the direct anti-tumor function of neutrophils. The PD-L1/PD-1 axis is extensively explored in the context of cancer, where tumor cell PD-L1 interacts with T cell PD-1 to limit adaptive anti-tumor immune responses (Figure 6a). However, in line with previous studies [15,16], we identified a PD-L1^pos^ subpopulation of neutrophils that is propagated in breast cancer. Thus far, the role of neutrophil PD-L1 was mainly reported in regard to the suppression of cytotoxic T cells (Figure 6b) [15]. Moreover, PD-L1^pos^ neutrophils were shown to contribute to the growth and progression in human tumors and are considered to be a poor prognostic factor indicative of reduced patient survival [16]. In line with these studies, we observed a high expression of PD-L1 on the non-cytotoxic tumor-infiltrating neutrophils (Figure 1c). In contrast, cytotoxic neutrophils isolated from the circulation or the premetastatic niche were relatively low in PD-L1 expression (Figure 1b,d). PD-L1^pos^ neutrophils exhibited a pro-tumor phenotype and could be mainly classified as immature LDN (Figure 1g). This observation provides further support, and possible mechanistic insight, into the immunosuppressive LDN phenotype [34]. Taken together, these observations suggest that PD-L1^pos^ neutrophils are characterized as having an immature, low-density, pro-tumor phenotype. The role of PD-L1-expressing neutrophils, in the context of T cell suppression and tumor growth, was already explored (Figure 6b). However, the role played by the PD-L1/PD-1 axis in regulating anti-tumor properties of neutrophils was unknown. We and others [30] have shown that a subpopulation of tumor cells express PD-1, which may facilitate interactions with PD-L1 expressed by other cells. Accordingly, we hypothesized that tumor cell PD-1 may interact with neutrophil PD-L1 and, thus, modulate neutrophil function. Indeed, blocking of the PD-L1/PD-1 axis in a co-culture setting enhanced tumor cell susceptibility to neutrophil cytotoxicity, suggesting that the PD-L1/PD-1 axis is involved in regulating direct neutrophil and tumor cell crosstalk. Moreover, this observation indicated that the PD-L1/PD-1 interaction in this context reduced the extent of neutrophil cytotoxicity (Figure 6c). Since PD-1 expression levels vary among different tumors, we speculated that the effect different tumors have on the function of PD-L1^pos^ may correlate with the extent of PD-1 expression. To test this idea, we overexpressed PD-1 in tumor cells and assessed tumor growth and progression in mouse models of breast cancer. This led to a more aggressive phenotype, as the tumors grew faster and had a higher metastatic load. Conversely, the knocking out of PD-1 resulted in reduced tumor growth and reduced metastatic colonization of the lungs. Furthermore, we showed that tumor cells overexpressing PD-1 were protected from neutrophil elimination at the premetastatic site. Importantly, these in vivo experiments were done using immunodeficient mice to eliminate the contribution of lymphocytes.

In summary, this study provides a novel insight into the interaction between neutrophil PD-L1 and tumor cell PD-1, and how this interaction regulates neutrophil anti-tumor cytotoxicity. The importance of PD-L1 and PD-1 on neutrophil cytotoxicity highlights the beneficial effect of blocking the PD-L1/PD-1 axis in cancer, not only to enhance T cell functions, but also to enhance neutrophil anti-tumor contribution. 

## Data Availability

Not applicable.

## Supplementary Materials

The following are available online at https://www.mdpi.com/article/10.3390/cells10061510/s1; Figure S1: PD-1 is not expressed in murine neutrophils.
