# Supplementary material for: The PD-L1/PD-1 Axis Blocks Neutrophil Cytotoxicity in Cancer
Source: Cells. 2021 Jun 15;10(6):1510. doi: 10.3390/cells10061510 (PMC8232689; doi:10.3390/cells10061510)
Supplement: Supplementary file 1 [file cells-10-01510-s001.zip › cells-1237168-supplementary.pdf]

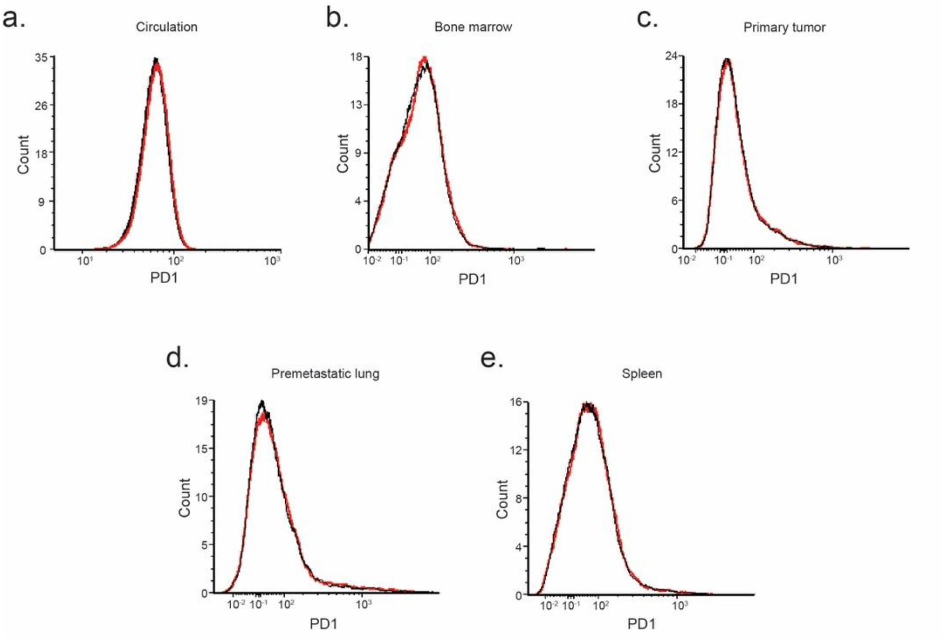

Figure S1: PD-1 is not expressed in murine neutrophils.
FACS analysis of PD-1 expression (red) in $Ly6G^+$ neutrophils isolated from the circulation (a), bone marrow (b), primary tumor (c), premetastatic lung (d) and spleen (e) of 4T1 tumor bearing mouse. Black histogram represents isotype control staining.
